# Cuidado em Saúde Baseado em Valor na Cardiologia: Como Integrar uma Visão mais Abrangente na Decisão Médica?

**DOI:** 10.36660/abc.20240668

**Published:** 2025-03-17

**Authors:** Pedro Gabriel Melo de Barros e Silva, Valter Furlan, Renato D. Lopes, André Volschan, Paulo Cesar Pereira de Souza, Carisi Anne Polanczyk

**Affiliations:** 1 Brazilian Clinical Research Institute São Paulo SP Brasil Brazilian Clinical Research Institute, São Paulo, SP – Brasil; 2 Hospital do Coração São Paulo SP Brasil Hospital do Coração (HCOR), São Paulo, SP – Brasil; 3 Hospital Samaritano Paulista São Paulo SP Brasil Hospital Samaritano Paulista, São Paulo, SP – Brasil; 4 Centro Universitário São Camilo São Paulo SP Brasil Centro Universitário São Camilo, São Paulo, SP – Brasil; 5 Hospital Ipiranga São Paulo SP Brasil Hospital Ipiranga, São Paulo, SP – Brasil; 6 Hospital Paulistano São Paulo SP Brasil Hospital Paulistano, São Paulo, SP – Brasil; 7 Duke Clinical Research Institute Duke University Medical Center Durham NC USA Duke Clinical Research Institute - Duke University Medical Center, Durham, NC – USA; 8 Hospital Pró-cardíaco Rio de Janeiro RJ Brasil Hospital Pró-cardíaco,Rio de Janeiro, RJ – Brasil; 9 Instituto Américas Rio de Janeiro RJ Brasil Instituto Américas, Rio de Janeiro, RJ – Brasil; 10 Faculdade de Medicina Universidade Federal do Rio Grande do Sul Porto Alegre RS Brasil Faculdade de Medicina - Universidade Federal do Rio Grande do Sul, Porto Alegre, RS – Brasil; 11 INCT para Avaliação de Tecnologia em Saúde Hospital Moinhos de Vento Porto Alegre RS Brasil INCT para Avaliação de Tecnologia em Saúde - Hospital Moinhos de Vento, Porto Alegre, RS – Brasil

**Keywords:** Qualidade da Assistência à Saúde, Seguro de Saúde Baseado em Valor, Doenças Cardiovasculares

## Abstract

O cuidado em saúde baseado em valor representa um conceito centrado no paciente e que, ao mesmo tempo, proporciona uma visão ampla de todas as partes envolvidas no processo assistencial. Este modelo visa o melhor desfecho possível, a um custo sustentável. Na sua descrição clássica, o cuidado baseado em valor é a relação entre desfechos que importam para o paciente e os custos necessários para alcançá-los. Posteriormente, outras variáveis foram consideradas nesta equação, como apropriabilidade e experiência do usuário, para tornar este modelo mais replicável. Ao trazer uma visão que alinha os interesses dos pacientes, dos médicos e das fontes pagadoras, a adoção do cuidado em saúde baseado em valor apresenta grande potencial para uma melhora sustentável da prática médica. Após mais de uma década de discussões e publicações, a aplicação desse conceito ainda representa um tema desafiador e dinâmico, o qual tem crescido em importância com o objetivo de se tornar um modelo transformador dos ecossistemas de saúde. A remuneração médica alinhada a esses princípios parece ser um caminho promissor para tal transformação, entretanto, a aplicação deste conceito depende de uma definição mais objetiva dos parâmetros relacionados ao valor em saúde nas diferentes situações clínicas. A cardiologia é uma especialidade que apresenta habitualmente uma base sólida de evidências para decisão médica, o que gera grande potencial para aplicação dos elementos de valor em saúde. Neste artigo, os autores apresentam uma revisão sobre os principais conceitos do cuidado em saúde baseado em valor e possíveis aplicações na cardiologia.

## Graphical abstract

**Figura Central:**
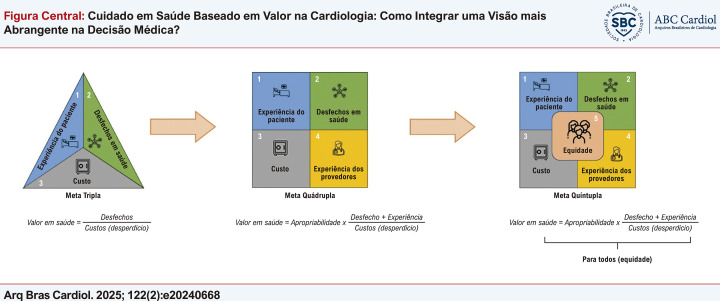
Cuidado em Saúde Baseado em Valor na Cardiologia: Como Integrar uma Visão mais Abrangente na Decisão Médica?

Fórmula clássica do valor em saúde e suas variações.

## Introdução

O conceito de Cuidado em Saúde Baseado em Valor (CSBV), do inglês *Value-Based Health Care* ou VBHC, foi introduzido de maneira formal no início dos anos 2000 e representa oferecer o melhor desfecho possível ao paciente de forma sustentável economicamente, visando a redução de desperdícios.^1 - 3^ É uma base conceitual para a reestruturação dos sistemas de saúde com o objetivo abrangente de oferecer cuidados de acordo com os valores que importam aos pacientes e que também estejam alinhados aos interesses de todas as partes envolvidas.^1 - 3^ Tendo em vista o constante aumento dos gastos em saúde sem que haja evidências de melhoria nos desfechos da população, fez-se necessário buscar estratégias para estimular a geração de melhores resultados com os recursos existentes dentro da capacidade do próprio sistema de saúde.^4 , 5^ Embora este modelo continue sendo pouco compreendido e mal mensurado na prática médica, já existem demonstrações de resultados efetivos de sua aplicação.^6 - 10^ Entretanto, ainda há aspectos que devem ser considerados ou aprimorados para que, de fato, haja aplicação em larga escala da assistência médica baseada em valor.

O presente artigo tem como objetivo discorrer sobre os conceitos de valor em saúde e suas aplicações práticas na cardiologia, além de apresentar um modelo de aplicação deste conceito.

### O que é valor?

O conceito de valor é algo, de certa forma, abstrato, mas que é utilizado para diversas decisões do nosso cotidiano. Na visão das ciências econômicas, “valor” representa a propensão a pagar por algo, ou seja, se o benefício recebido pelo cliente supera o custo a ser pago por aquele produto ou serviço.^11 , 12^ Em saúde, o benefício deve estar centrado no paciente e, essencialmente, relacionado a uma melhoria na qualidade de vida, redução da “carga” da doença e/ou aumento da sobrevida. O custo deve ser calculado pelo consumo de recursos relacionados ao cuidado do paciente, o qual pode ser avaliado de forma global (custos diretos e indiretos) ou de forma específica (custo direto de uma intervenção a ser realizada); estes gastos também podem ser calculados em diferentes perspectivas (paciente, plano de saúde).^13^ A relação entre o benefício do paciente e o custo do cuidado fundamenta o racional do que seria “valor” em saúde.

### Conceito original de valor em saúde

O conceito de “valor nos cuidados em saúde” foi apresentado de maneira mais objetiva em publicações do professor Michael Porter da escola de negócios de Harvard.^1 , 2^ Na equação original sobre cuidado baseado em valor ( Figura Central ), o conceito pode ser interpretado da seguinte maneira: quanto melhor o desfecho sob a perspectiva do paciente e quanto menor o custo da ação médica, maior o valor oferecido.^2^ Além da assistência médica, este conceito intuitivo de “valor” é aplicado em várias decisões da vida cotidiana, como comprar um bem material ou contratar um serviço, pois quanto melhor o resultado da aquisição (satisfazer as necessidades de forma duradoura) e menor o custo, melhor será a aplicação dos recursos e, por consequência, maior será o valor atribuído a essa escolha.

Embora simples e intuitivo, existem pontos de atenção na aplicação deste conceito para as atividades em saúde. A preocupação de que o uso da fórmula de valor em saúde possa recompensar resultados ruins que são alcançados a baixo custo é algo pertinente e que deve ser considerado de forma cuidadosa quando da implementação desse paradigma.^1 - 3 , 12^ Dessa forma, as duas variáveis da equação devem ser consideradas também de forma separada e não apenas de forma conjunta, uma vez que não existe uma métrica universal de valor que se baseia na relação das duas variáveis (desfecho/custo) e que possa ser aplicada em todas as decisões em saúde.^1 - 3 , 14^ Além disso, este conceito de valor foi criado para ser aplicado apenas quando não há penalização dos desfechos clínicos relevantes, ou seja, custos mais baixos geram real valor quando os resultados obtidos pelos pacientes são mantidos ou melhorados.^1 - 3^

#### Desfechos

Os desfechos representam impactos em saúde percebidos pelos pacientes e gerados por processos coordenados.^14 - 17^ Embora a mensuração de indicadores de processos (ex: tempo porta-balão no infarto agudo do miocárdio) seja uma ação importante na melhoria assistencial, este tipo de métrica se traduzirá em valor em saúde se ele resultar em melhores indicadores na perspectiva do paciente, como maior sobrevida, qualidade de vida e redução de complicações. Os indicadores de processos devem ser perseguidos para assegurar qualidade assistencial e planejamento tático dos serviços, mas eles não devem ser considerados como objetivo final nas entregas em saúde. Nesse sentido, há variação de desfechos de acordo com as doenças e entre indivíduos, sendo importante buscar os desfechos que tenham maior relevância e validade na saúde populacional, e que também possam ser avaliados de forma objetiva e hierárquica (ex: numa cirurgia de próstata a hierarquia pode ser sobrevivência, incontinência e disfunção erétil, embora essa ordem possa sofrer variações entre indivíduos). É fundamental que os desfechos representem benefícios sustentados para o paciente e que sejam relevantes também para as outras partes interessadas no cuidado à saúde.^14^

Além dos desfechos clínicos propriamente ditos, vale destacar que há diversas ações que podem influenciar o custo do cuidado em saúde e/ou a experiência dos indivíduos, mas não necessariamente o desfecho clínico do paciente. Tais processos coordenados embora não alterem o desfecho clínico, podem reduzir o custo e o desperdício e, caso não comprometam sobrevida e qualidade de vida, poderiam aumentar o valor em saúde.

#### Custos

Na análise do valor em saúde, uma vez que os desfechos não sejam comprometidos (numerador), qualquer ação que implique em menores custos (denominador), por consequência, gera maior valor.^2^ Nesse modelo de raciocínio, a organização de processos assistenciais que agilizem o fluxo de atendimento com métricas bem definidas para melhoria contínua, mesmo que não mude o desfecho, pode melhorar a eficiência (e eventualmente a experiência dos indivíduos) e, por consequência, reduzir o custo e aumentar o valor em saúde.^18^ O custo inicialmente é calculado de forma direta, entretanto, pode-se considerar a inclusão de custos indiretos no denominador da fórmula de valor em saúde para uma avaliação mais abrangente.

No Brasil ainda há muitos desafios para estimar os custos reais do cuidado em saúde, o que muitas vezes gera a situação em que para um procedimento específico, o qual deveria ter um custo semelhante em diferentes unidades, terá pagamentos com valores diferentes (variação de acordo com o tipo de plano de saúde ou negociação do hospital). A lógica financeira da maioria das instituições nacionais é baseada em unidade estrutural (unidade de terapia intensiva, emergência, hemodinâmica), com distribuição de valores por técnicas de rateio ou absorção, não considerando a trajetória do paciente ou uma determinada condição de saúde. As técnicas de microcusteio permitem estimar o custo real dos serviços em saúde.^19 , 20^Essa distorção se reflete tanto no sobrefinanciamento em saúde (preço muito acima do custo) como no subfinanciamento em saúde (valor repassado inferior ao custo do atendimento). A coexistência destes dois extremos gera uma situação de custos não controlados que estressam a sustentabilidade do sistema de saúde.

## Conceito de apropriabilidade

Além de desfecho e custo, outras duas variáveis têm sido discutidas nesta equação: apropriabilidade^21 , 22^ e experiência do paciente.^22^

Apropriabilidade (ou pertinência) indica se alguma ação médica apresenta embasamento científico adequado para melhora do desfecho do paciente.^21 , 22^ Numa análise categórica (sim ou não), esta variável entraria na fórmula como 1 ou 0, ou seja, se for apropriada (melhora desfecho), a quantificação é feita de acordo com a fórmula clássica (impacto no desfecho/custo), entretanto, se não for apropriada, o resultado sempre será zero (não haverá valor). Esse conceito já é considerado nas análises de custo-efetividade em que, se uma ação não é eficaz ou efetiva, não existe motivo para avaliar o custo pois, por menor que ele seja, esta ação não será custo-efetiva.^23^

Entretanto, em muitas decisões médicas há uma zona “cinza” em que não seria possível atribuir um valor 0 ou 1 (especialmente em parâmetros populacionais numa situação clínica heterogênea).^24^ Tendo em vista a existência de situações de incerteza quanto à apropriabilidade, essa variável é especialmente útil para aplicações nos casos em que há evidência suficiente para categorizar uma decisão como apropriada ou não apropriada. Esse é um modelo simples que pode ser aplicado de forma objetiva em ações que claramente não são apropriadas e que, por mais que o paciente tenha um bom desfecho com “baixo custo”, não geram valor (uma vez que este mesmo desfecho seria alcançado com “custo zero”).^23 , 24^ A identificação dessas ações sem pertinência permite evitar o desperdício que ocorre em ações de sobrediagnóstico e de sobretratamento ( Quadro 1, material suplementar ). De qualquer maneira, mesmo em situações de zona “cinza” (ex: cenários que não são exatamente aqueles testados em estudos clínicos), ter esta visão do valor em saúde irá levar o médico e/ou gestor a buscar mais elementos para tomar decisões de forma adequada aos pacientes, uma vez que reconhece estes cenários como situações de apropriabilidade e valor incertos.

Apesar do racional para considerar a “incerteza” sobre a apropriabilidade em algumas situações, geralmente, as recomendações internacionais (americanas, europeias) consideram que a ação médica que não possa ser classificada como apropriada, já seria, intrinsecamente, considerada como parte do desperdício e má-prática.^21 - 27^ Isto se baseia particularmente nas consequências potenciais do sobrediagnóstico e sobretratamento, as quais podem ser significativas e incluem desde efeitos psicológicos e comportamentais da estigmatização de pacientes até danos físicos/efeitos colaterais de testes ou tratamentos desnecessários que prejudicam a qualidade de vida e que podem, inclusive, abreviar a sobrevida do indivíduo.^21 - 27^ Além da possibilidade de piorar os desfechos, estas ações excessivas elevam os custos financeiros individualmente e geram desperdício de recursos e de oportunidades no sistema de saúde. Apesar de conceitualmente fazer sentido a não realização de procedimentos sempre que houver dúvidas sobre o seu real benefício, por outro lado, é importante também questionar se a não execução desses procedimentos não iria gerar a consequência inversa, ou seja, não iria ocasionar subdiagnóstico ou subtratamento. Embora este equilíbrio seja desafiador, deve-se ter em mente que a aplicação do conceito do valor em saúde é um caminho racional para atingir a melhor maturidade nas decisões médicas e de políticas de saúde.

## Outras metas no cuidado em saúde

Além de desfecho, custo e apropriabilidade, outras métricas devem ser consideradas também como metas no cuidado baseado em valor. Embora a inclusão da experiência do paciente (“cliente”) na fórmula de valor tenha grande apelo do ponto de vista de mercado em saúde, isto pode trazer mais “ruído” do que informação sobre o resultado clínico propriamente dito.^1 , 2 , 15^ Isso não quer dizer que a experiência não agrega valor no cuidado do paciente, entretanto, trata-se de um outro tipo de valor (ex: percepção do usuário que inclui variáveis como hotelaria, recepção, serviços auxiliares da unidade de saúde, dentre outros). Apesar de representar algo diferente do desfecho propriamente dito, a experiência do paciente nos cuidados em saúde deve ser valorizada como os outros elementos do CSBV, especialmente na busca do que, de fato, importa ao paciente e que possa ser mensurado de forma confiável.^28^

Uma vez acrescida esta variável (experiência do paciente) na valoração dos serviços, é importante que isto seja avaliada de forma independente dos desfechos. Por exemplo, um paciente pode ter sido submetido a um tratamento de alto custo que não mudou o seu desfecho pois era uma terapia inapropriada (sobretratamento) e, por mais que a experiência tenha sido boa, não houve valor em saúde (o contrário também é verdadeiro, ou seja, um tratamento apropriado que melhorou o desfecho clínico com baixo custo gerou alto valor em saúde mesmo que o paciente não tenha ficado satisfeito com o acolhimento da equipe, por exemplo). Dessa forma, os problemas relacionados à experiência devem ser reconhecidos e manejados de forma específica (ou seja, de forma distinta em relação ao desfecho clínico).

Finalmente, outros itens têm sido agregados nos objetivos primários do cuidado em saúde, desde a fórmula clássica de valor em saúde que se baseava apenas em desfechos e custos. A chamada tripla meta (do inglês, *triple aim* ), indica que o sistema de saúde deve ter foco não só no desfecho do paciente a um custo sustentável, mas também na experiência do usuário.^22^ Este conceito evoluiu para meta quadrupla (do inglês, *quadruple aim* ) quando, além de considerar a experiência do paciente, também inclui a do profissional de saúde. Assim, além de buscar o melhor desfecho para o paciente a um menor custo possível, visa-se igualmente proporcionar a melhor experiência tanto para o paciente quanto para o profissional de saúde.^25^

Mais recentemente, incluiu-se a equidade na saúde também como um dos objetivos primários da agora chamada meta quíntupla (do inglês, *quintuple aim* ).^29^ Neste caso, busca-se reduzir as disparidades de acesso e de resultados dos cuidados de saúde entre diferentes populações, removendo barreiras geográficas, financeiras ou culturais em comunidades desfavorecidas ( Figura Central ).^29^ Estes conceitos podem ser melhor aplicados em redes de saúde, as quais, ao realizarem a gestão de uma população ampla e diversa, podem atuar de forma mais robusta na equidade do cuidado.^29^ Neste sentido, deve haver a integração da prestação de cuidados nas múltiplas unidades de saúde não só para reduzir custos (ex: evitar a duplicação de esforços, atrasos e outras ineficiências no processo de saúde), mas também para real gestão populacional e, finalmente, melhora da equidade.^30^

## Aplicações do conceito de valor em saúde na cardiologia

O desafio atual tem sido transformar os aspectos conceituais em ações práticas, proporcionando maior compreensão para cada um dos “atores” dentro desse novo modelo. Embora o CSBV seja centrado no paciente e dependa de um cuidado baseado em time para implementação e sucesso deste modelo,^31^ algumas decisões da rotina do cardiologista podem ser pautadas pelos princípios do valor em saúde. Abaixo são explorados exemplos para aplicação destes conceitos na doença coronariana:

## Síndrome coronariana aguda (SCA)

Embora, muitas vezes, o modelo de valor em saúde seja aplicado em intervenções terapêuticas (ex: cirurgias),^32 , 33^ este conceito também pode ser considerado em outras ações médicas como, por exemplo, no diagnóstico de infarto agudo do miocárdio. O biomarcador de escolha para este diagnóstico é a troponina de alta sensibilidade,^34^ entretanto, a CKMB continua sendo solicitada rotineiramente de forma concomitante à troponina em muitos serviços.^35^ Utilizando a fórmula de valor expandida, pode-se ver que a adição de dosagem rotineira de CKMB não agrega valor ao cuidado do paciente pois não tem impacto positivo em nenhuma das variáveis: 1) não é considerado um exame apropriado, 2) não contribui para a melhoria do desfecho do paciente, 3) aumenta o custo, 4) e se for analisar a experiência do paciente, um exame adicional não lhe traz benefícios. Dessa forma, embora não sejam utilizados números nessa fórmula, pode-se ver claramente que não há valor nesta ação médica.

Em um ambiente de atendimento de doença tempo-sensível como a SCA com elevação de ST, a avaliação por um médico especialista seria o “padrão-ouro” para diagnóstico e conduta. Entretanto, é inviável ter um cardiologista disponível em todas as emergências para atendimento destes pacientes. Desse modo, o apoio de um especialista via telemedicina seria uma estratégia atrativa para ter o suporte do cardiologista em um maior número de unidades. Este modelo demonstrou se associar a aprimoramentos na terapia dos pacientes com SCA e, por consequência, melhora nos desfechos.^36 , 37^ Embora as evidências específicas sobre a telemedicina neste cenário de SCA ainda careçam de validação em ensaios clínicos randomizados, trata-se de uma ação que foi idealizada com o princípio da entrega de valor em saúde, ou seja, melhorar desfechos a um custo sustentável.

A oferta de valor em saúde começa com foco na qualidade (desfecho), e diversos estudos randomizados têm demonstrado de forma consistente que intervenções de melhoria de qualidade geram maior uso de terapias baseadas em evidência e alguns possuem impacto suficiente para demonstrar melhores desfechos.^38 - 41^ Especificamente em SCA, um estudo nacional chamado BRIDGE-ACS^38^ avaliou de forma randomizada o impacto de intervenções de qualidade assistencial. Embora não tenha sido delineado com poder para avaliar impacto em desfechos clínicos, o BRIDGE-ACS demonstrou melhora significativa no uso de terapias baseadas em evidência em SCA.^38^

O cuidado baseado em valor tem a melhora da qualidade assistencial e dos desfechos clínicos dos pacientes como seu objetivo prioritário. Entretanto, a entrega de valor deve também focar no custo envolvido para obter este desfecho desejado, ou seja, ambos (desfechos e custos) devem ser sempre avaliados. Em uma experiência nacional, observou-se que a utilização de ferramentas internacionais para ações de qualidade assistencial se associou à melhora no tratamento e nos desfechos dos pacientes com SCA.^42^ Entretanto, havia necessidade de entender se essas ações também iriam causar impacto no custo e, quando analisado os custos hospitalares e taxas de reinternação de pacientes com SCA, foi observado que o custo hospitalar foi melhor controlado e com menos reinternações no hospital sob as ações de melhoria de qualidade assistencial quando comparado ao custo de pacientes semelhantes em outras unidades.^43^ Conforme demonstrado neste exemplo em que métricas de qualidade e de custo foram aferidas em um programa voltado para a melhora da qualidade assistencial em SCA,^42 , 43^ é de se esperar que, nas difererentes linhas de cuidado cardiológicas, uma vez tendo melhores desfechos (menos complicações), o custo em saúde também seja menor e que haja menos reinternações.

## Revascularização miocárdica

Nos pacientes em que há indicação apropriada de revascularização miocárdica, ou seja, que existem evidências de melhores desfechos para o paciente com esta estratégia, é importante avaliar qual procedimento traria melhor valor em saúde de forma sustentada.^44^

Nas situações em que houver indicação apropriada de revascularização miocárdica, seja percutânea ou cirúrgica, deve-se também avaliar como melhorar o custo e a experiência do paciente sem comprometer o desfecho clínico. De uma forma geral, ações que melhoram a qualidade, aumentarão o valor não apenas pelo impacto em melhores desfechos mas também porque a melhor qualidade em revascularização miocárdica se associa a menores custos.^44 , 45^ Dessa maneira, além das ações focadas para obter o melhor desfecho clínico, iniciativas para aprimorar a eficiência (ex: para proporcionar redução de tempo de internação pós-cirurgia cardíaca e pós-angioplastia), também agregam valor ao processo assistencial, uma vez que reduzem os custos sem comprometer os desfechos clínicos (podendo inclusive melhorar a recuperação do paciente).^46 , 47^ Iniciativas como os protocolos de alta no mesmo dia após angioplastia não complicada^46^ e de rápida recuperação pós-cirurgia (ERAS, do inglês, *enhanced recovery after surgery* ),^47^ embora ainda não tenham evidências robustas para recomendações mais amplas, são ações que visam oferecer valor, pois buscam pelo menos manter os desfechos clínicos e, ao mesmo tempo, reduzir custos e melhorar a experiência do paciente.

## Outros exemplos

Qualquer que seja a situação clínica (intervenção, doença crônica), a entrega de valor depende de uma rede assistencial integrada em que o atendimento é prestado por uma equipe multidisciplinar dedicada, a qual assume a responsabilidade pelo ciclo completo de atendimento a uma condição específica, abrangendo atendimento ambulatorial, hospitalar e de reabilitação, além de serviços de apoio.^30 , 48 , 49^ A adoção das melhores práticas deve ser guiada por diretrizes baseadas em evidência^50 , 51^ e, além dos exemplos de aplicação pontual dos conceitos de valor em saúde em SCA, revascularização miocárdica, há outros exemplos na cardiologia em que há uma implementação mais ampla deste novo modelo. Nesta aplicação plena do cuidado baseado em valor, há necessidade de criar-se uma forma de remuneração compatível, uma vez que os modelos de remuneração atuais não estão alinhados com os princípios do cuidado baseado em valor.^5 , 52^

## Modelos tradicionais de remuneração

Atualmente, há diferentes modelos de remuneração ( Quadro 2, material suplementar ) aplicados nos diversos sistemas de saúde, muitos dos quais coexistem dentro um mesmo ecossistema.^53 - 55^

- A forma de remuneração mais antiga e vigente nos sistemas de saúde, incluindo o Brasil é o pagamento por serviço ( *fee-for-service* ), o qual foca em reembolso baseado na produção de atividades e, dessa forma, não alinha os interesses de todas as partes envolvidas ( Quadro 2, material suplementar ). Neste modelo, ao utilizar um plano de saúde, tanto o usuário (paciente) como o prestador de serviços (profissional de saúde ou instituição) estabelecem uma relação em que as entregas estão vinculadas ao produto (ou serviço prestado) e não ao real ganho (ou benefício) em saúde. Ou seja, independentemente dos resultados em saúde ou pertinência do serviço, haverá um pagamento fixo, o qual incentiva a produção em maior quantidade, mesmo que isso não gere efetivamente valor em saúde. Neste modelo, estão inseridas formas de pagamento usualmente designadas por remuneração por unidade de serviço ou conta aberta, e remuneração por pacotes de procedimentos e diárias hospitalares, tendo como essência, o pagamento por número de procedimentos individuais. Uma das principais desvantagens deste modelo é o estímulo à utilização de serviços em grupos de benefício questionável, principalmente os que proporcionam margens de lucro mais elevadas e que, portanto, podem afetar negativamente a qualidade da atenção à saúde e a sustentabilidade do sistema. Atualmente, este é o modelo mais associado na literatura ao aumento desnecessário no custo da assistência médica.
- O modelo por “pacote” ( *bundles* ) consiste em agrupar o pagamento por determinada condição de saúde, procedimento ou cirurgia considerando um período longitudinal de acompanhamento. Pode ou não considerar compartilhamento de risco entre pagadores e prestadores. Há “pacotes” específicos (ex: para condições cardíacas agudas, pós-cirurgia ou intervenção transcateter), com acompanhamento geralmente de 30 a 90 dias. O pagamento por “pacote” também pode dar maior retorno financeiro pelo volume e, além disso, quando os custos das complicações são assumidos pela própria equipe, ele também estimula a realização de procedimentos nas situações com menor probabilidade de complicações. Isto é um risco potencial para a realização de procedimentos em situações com menor necessidade (ou apropriabilidade) e, além disso, as equipes tenderiam a evitar procedimentos que, mesmo sendo apropriados, apresentem risco considerável de complicações (o que pode gerar maior limitação do cuidado nos grupos de maior gravidade).
- No modelo per capita ou de capitação ( *capitation* ) os provedores dos sistemas de saúde recebem uma remuneração fixa para o cuidado parcial ou integral da saúde de uma população definida. O modelo de capitação tem o conceito de assegurar o cuidado necessário para quem precisa, independente se essa pessoa utiliza ou não o serviço. O valor é estabelecido por pessoa incluída no programa (per capita) e a cada período. Dessa forma, o valor da remuneração é baseado na média esperada de utilização de cada paciente (remuneração planejada deverá variar de acordo com idade, raça, sexo, região e, principalmente, histórico médico). Esses modelos podem ter suas bases de relação considerando entregas objetivas por serviço prestado (ex: número de pacientes hipertensos com pressão arterial aferida ou colonoscopia realizada em indivíduos acima de 50 anos) ou por métricas mais amplas que se referem a indicadores de saúde populacional (mortalidade por infarto do miocárdio, internação por insuficiência cardíaca). Deve-se destacar que essa modalidade pressupõe o conhecimento dos riscos à saúde associados à cobertura da população definida, bem como a contrapartida em termos de custos.^56^ Uma das grandes dificuldades no avanço do modelo de capitação é que o seu sucesso depende de uma cadeia de análise mais prolongada (com tempo e variáveis a serem consideradas) uma vez que há o risco potencial de se restringir o acesso ao cuidado em saúde para reduzir o custo no curto prazo e, por consequência, postergar o problema do paciente para mais adiante (o que pode aumentar o custo a longo prazo). Dessa forma, a segurança na sustentabilidade deste modelo depende de uma boa gestão de informações dos cuidados, desfechos e custos dos pacientes para evitar, principalmente, situações de subdiagnóstico e subtratamento decorrentes de uma eventual restrição de acesso no curto prazo.

## Remuneração baseada em valor

A remuneração ou pagamento baseado em valor representa um modelo de prestação de cuidados em saúde no qual os prestadores, incluindo hospitais e médicos, são pagos com base nos resultados de saúde dos pacientes ( Quadro 2, material suplementar ). Neste tipo de acordo, os provedores assumem a responsabilidade pelas entregas em saúde aos pacientes. A remuneração baseada em valor apresenta um estímulo mais adequado do que os outros modelos vigentes uma vez que tem como princípio o melhor desfecho do paciente ao menor custo possível.^54^ Por consequência, diferente de outros modelos em que o retorno financeiro se vincula a um maior número de serviços ( *fee for service* ) ou reduzindo procedimentos de maior custo/risco ( *bundles, capitation* ), a remuneração baseada em valor se baseia eminentemente no desfecho do paciente e, por consequência, há alinhamento de interesses de todas as partes envolvidas.^55^

Ao usar um modelo de remuneração baseado em valor, é importante entender que parte do desfecho depende do paciente (ex: adesão medicamentosa) e não diretamente dos profissionais de saúde. De qualquer forma, a adesão do paciente é diretamente relacionada com a educação em saúde e, neste caso, ter um modelo que realiza análises comparativas de resultados entre profissionais ou equipes pode melhorar o resultado como um todo sem descaracterizar a remuneração baseada em valor.

Nesse modelo integrado e coordenado, os provedores de saúde trabalham como uma equipe em rede para fornecer o melhor atendimento possível a um custo necessário para alcança-lo. Dessa maneira, além do resultado individual, a equipe da linha de cuidado compartilha o risco e o retorno financeiro. A linha estruturante do modelo de pagamento baseado em valor deve focar na adoção das melhores práticas médicas, baseadas em evidências científicas e diretrizes internacionais, com um horizonte temporal de longo prazo de resultados.^54 , 55^

Outro modelo alternativo de pagamento é a Orçamentação Global ou Transferência Orçamentária, que é uma forma de remuneração prospectiva, na qual o estabelecimento de saúde estima, anualmente, suas necessidades de gastos e apresenta ao ente financiador e, em contrapartida, compromete-se com o cumprimento de metas de desempenho, baseadas em métricas de eficiência e qualidade da prestação de serviços.^57^ Uma das principais questões relativas a este modelo é a complexidade de sua implementação, e mensuração de indicadores, quando existem múltiplos pagadores de serviços vinculados àquele estabelecimento.

## Situação atual dos modelos de remuneração

As atuais reformas em andamento nos sistemas de saúde no mundo têm a pretensão de reduzir a amplitude do modelo vigente do pagamento por serviço ( *fee-for-service* ) e substituí-lo progressivamente por modelos alternativos de remuneração.^53 - 58^ No contexto internacional, especificamente no mercado de seguros e planos privados de saúde, têm-se assistido ao surgimento de novos produtos, como o que se convencionou chamar no mercado americano de *Value Based Insurance Products* .^58^ De um modo geral, tanto a iniciativa dos *Shared Savings Program* (SSP) quanto as dos *Bundled Payments* podem ser agrupados dentro da categoria dos novos modelos de remuneração e compartilhamento de risco, surgidos nas reformas internacionais e que são orientados por valores de qualidade e eficiência.

## Cultura da medicina baseada em valor

A implementação de um modelo de pagamento totalmente diferente do vigente, num sistema complexo como o de saúde, depende não apenas das ações dos gestores em saúde, mas também de uma mudança cultural de médicos, pacientes e sociedade em geral.

O valor em saúde é determinado pela relação entre os resultados clínicos alcançados e o custo necessário para obtê-los. Esse conceito não deve ser restrito ao médico e ao gestor de saúde, mas deve fazer parte da decisão do paciente. Assim como na aquisição de bens e contratação de serviços de forma geral, o “cliente” em saúde também deveria buscar informações dos resultados de um hospital ou médico. No modelo atual de assistência em saúde, muitas vezes, os profissionais e hospitais são escolhidos pelo custo (baixo ou elevado) e/ou reconhecimento social, como se fossem sinônimos de valor em saúde, o que não necessariamente assegura o conceito descrito.

Há uma assimetria de informação em que os pacientes têm poucas informações sobre aspectos fundamentais, como: apropriabilidade das ações médicas, resultados clínicos (do médico e do hospital) e custos evitáveis no cuidado em saúde. A disponibilidade destas informações, além de beneficiar o paciente, pode gerar também redução do custo em saúde o que, em última análise, poderá aumentar o acesso de pacientes ao sistema de saúde e elevar a remuneração média dos profissionais num modelo de pagamento baseado em valor.

Uma das grandes dificuldades para implementação desses modelos mais adaptativos é garantir a segurança e veracidade das informações reportadas por prestadores (hospitais, clínicas, profissionais), o que ainda limita a aplicação deste conceito de forma mais plena apenas em sistemas de saúde mais controlados (ex: redes verticalizadas).^59^

Seguindo os preceitos do cuidado baseado em valor, surgiram as *Accountable Care Organizations* (ACOs), que são organizações as quais o paciente e os provedores são verdadeiros parceiros nas decisões de cuidado (centradas no paciente).^60^ Neste modelo, há ênfase na coordenação do cuidado com o compartilhamento de dados clínicos e de sinistralidade entre os membros da equipe e também com os pagadores para que a remuneração esteja vinculada a melhorias nos resultados (como readmissões hospitalares, eventos adversos, envolvimento do paciente e indicadores de saúde da população). Para que seja de fato considerado um modelo baseado em valor, ele deve ter como princípio o melhor desfecho ao paciente, com a cautela em não perseguir somente o resultado econômico. Métodos para avaliação uniforme de desfechos têm sido divulgados e aplicados como o ICHOM ( *International Consortium for Health Outcomes Measurement* ), os quais têm sido úteis na condução de linhas de cuidado.^61^ Modelos de coleta diretamente do paciente tanto para desfechos (PROMs, do inglês, *Patient-Reported Outcomes Measures* ) como para experiência (PREMs, do inglês, *Patient-Reported Experience Measures* ) são recomendados.^62^

De qualquer forma, seja numa aplicação em maior ou menor escala, a cultura do valor em saúde deve estar presente no delineamento dos serviços médicos tendo como princípio ações que de fato proporcionem o melhor desfecho através de um processo coordenado de qualidade e segurança assistencial. Uma vez definido e pactuado o que configura melhores desfechos para determinadas condições de saúde ou populações, estes processos devem ser avaliados quanto à possibilidade de serem mais eficientes e, por consequência, reduzir custos para entrega de maior valor em saúde. Um dos passos iniciais para aplicação na prática seria reconhecer as barreiras e buscar soluções de forma contínua e adaptada a cada realidade ( Quadro 3, material suplementar ).

## Proposta para aplicação do conceito de valor em saúde

**Figura 1:**
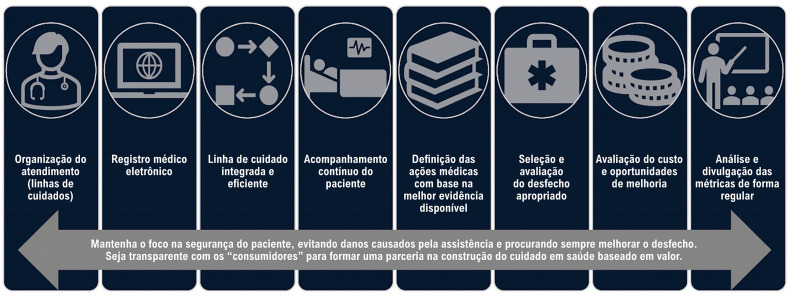
– Elementos fundamentais para cuidado em saúde baseado em valor (framework).

Em muitos locais, a implementação do modelo de CSBV tem sido feita em projetos pilotos para que sejam realizados ajustes iniciais antes de expandir. Nestes serviços, habitualmente as equipes possuem boa comunicação, bom conhecimento de medicina baseada em evidências, dados confiáveis e boa adesão a protocolos.^63 - 65^ Seguem alguns elementos fundamentais para um sistema baseado em valor ( Figura 1 ):

- Organizar o atendimento de acordo com as condições médicas, ou seja, linhas de cuidados para condições médicas dos pacientes ou segmentos da população;
- Proporcionar registros médicos eletrônicos que permitam compartilhamento de informações entre todos os prestadores da linha de cuidado;
- O atendimento deverá se basear num modelo em “time” com compartilhamento de dados do paciente para um processo coordenado que reduza cuidados redundantes (e custos associados) e que inclua desfechos de fácil aferição (nos modelos de saúde baseados em valor, o atendimento médico em emergência, atenção primária ou especializada não ocorre em “silos” e, sim, numa linha de cuidado integrada e centrada no paciente);
- Manter o paciente em acompanhamento de forma contínua, proporcionando uma adequada coordenação e entrega do cuidado;
- Definir a apropriabilidade das ações médicas de acordo com a situação médica com base na melhor evidência disponível;
- Uma vez definida a apropriabilidade, avaliar as métricas a serem aferidas (desfechos que realmente importam para o paciente de acordo com o tipo de doença e tratamento proposto);
- Ter métricas não apenas de desfecho clínico, mas também de custo para cada paciente e avaliar as oportunidades de melhoria da eficiência e da experiência do paciente;
- Avaliar os indicadores de desfecho de forma regular e divulgá-las a toda equipe assistencial;
- Manter o foco na segurança do paciente (evitar danos causados pela assistência) e procurar sempre melhorar o desfecho clínico;
- Por fim, ser transparente com os “consumidores” para que possam ser parceiros na construção do CSBV.

A implementação de pagamento baseado em valor deverá ser feita de acordo com as premissas acima e, embora ainda não seja exista uma fórmula universal, deve-se buscar modelos de pagamentos que recompensam os melhores desfechos (relevantes e sustentados) e a eficiência do atendimento (menor custo possível desde que não comprometa o desfecho). Do ponto de vista de sistema de saúde, uma forma de ter maior eficiência seria realizar a integração de uma rede assistencial em que a oferta de cuidados esteja organizada de forma hierárquica. O racional dessa integração tem como pressuposto o fato de que um sistema com vários centros que atendam casos de alta complexidade irá gerar um ambiente de menor eficiência, maior custo e, por consequência, menor valor. Além da maior eficiência e menor custo numa rede integrada hierárquica, a concentração de linhas de cuidado em unidades assistenciais de referência permite que, sob maior volume, o processo de qualidade se desenvolva mais plenamente e, dessa forma, são esperados melhores desfechos (o que aumentaria ainda mais o valor entregue aos pacientes).

## Conclusões

A mudança do sistema atual para um modelo baseado em valor é desafiadora, embora a transição já esteja acontecendo em diferentes partes dos ecossistemas de saúde. Este é um processo que tende a ser gradual, mas que deve ser ampliado rapidamente, especialmente no cuidado de pacientes crônicos. A cardiologia baseada em valor é uma tendência internacional para que seja possível aumentar o atendimento de qualidade com os menores custos possíveis. Esse pode ser o caminho para ajudarmos mais pessoas a terem vidas mais saudáveis em um sistema de saúde sustentável.

## *Material suplementar

Para informação adicional, por favor, clique aqui


