# Supplementary material for: Cuidado em Saúde Baseado em Valor na Cardiologia: Como Integrar uma Visão mais Abrangente na Decisão Médica?
Source: Arq Bras Cardiol. 2025 Mar 17;122(2):e20240668. [Article in Portuguese] doi: 10.36660/abc.20240668 (PMC12058157; doi:10.36660/abc.20240668)
Supplement: Supplementary file 1 [file 0066-782X-abc-122-2-e20240668-suppl01.pdf]

Quadro 1: Conceitos de falhas na utilização de recursos diagnósticos e terapêuticos[1-3]:

| **Tipo de falha na utilização de recursos diagnósticos e terapêuticos** | **Conceito** |
|---|---|
| Sobrediagnóstico (do inglês, *overdiagnosis*) | Situação em que um diagnóstico possa ser considerado "correto" nos padrões atuais, entretanto, este diagnóstico tem baixa probabilidade de beneficiar o paciente e pode, inclusive, gerar dano ao mesmo. Exames de checkup e rastreamento indiscriminado geram problemas relacionados tanto a achados "falso positivos" como, mesmo em diagnósticos corretos, o exame pode não trazer valor ao cuidado em saúde e apenas agregar custos, desconforto e outros inconvenientes e riscos relacionados a uma investigação diagnóstica desnecessária (sobrediagnóstico). |
| Sobretratamento (do inglês, *overtreatment*) | Termo que está intrinsecamente relacionado com o diagnóstico "excessivo" uma vez que um sobrediagnóstico gera habitualmente um sobretratamento subsequente. Esta intervenção também é considerada desnecessária quando apresenta baixa probabilidade de beneficiar o |

| | |
|---|---|
| | paciente podendo, inclusive, gerar dano ao mesmo. |
| Subdiagnóstico (do inglês, *underdiagnosis*) | Situação em que a não realização de uma investigação impossibilita um diagnóstico que traga benefício para o paciente. Esta situação deve ser sempre ponderada em decisões de políticas de saúde cujo alvo seja reduzir o sobrediagnóstico (cuidado para que tais ações de redução do sobrediagnóstico não gerem subdiagnóstico). |
| Subtratamento (do inglês, *undertreatment*) | Intrinsecamente ligada ao subdiagnóstico, uma vez que se não for feito um diagnóstico que traga benefício para o paciente, por consequência, perde-se a oportunidade do tratamento efetivo para o mesmo. |

Quadro 2. Modelos de remuneração em saúde

| Modelo de remuneração | Descrição (vantagens e desvantagens) |
|---|---|
| *Fee for service* | Pagamento por serviço (consulta, exame, intervenção).<br><br>Vantagens: modelo que poderia ser aplicado em casos cuja intervenção seria considerada apropriada mas que pacientes apresentam alta complexidade (baixa segurança no desfecho).<br><br>Desvantagens: remuneração do profissional depende exclusivamente de "volume", independente da apropriabilidade, dos desfechos do paciente e da eficiência do processo. |
| *Capitation* | Pagamento por "vidas"<br><br>Vantagens: oferece segurança financeira tanto para provedores (médicos, hospitais) quanto para pagadores (seguradoras). O melhor cuidado do paciente reduz a necessidade de atendimento médico o que aumenta o ganho monetário por atendimento do paciente nesses casos.<br><br>Desvantagens: os prestadores de serviços assumem o risco de atender mais pacientes do que o esperado mesmo que seu tratamento seja adequado; essa variabilidade não deve exceder a capacidade para atendimento com qualidade. |
| *Bundles* | Pagamento por internações com valor fixo ("pacote"). |

| | |
|---|---|
| | Vantagens: modelo que estimula o melhor desfecho hospitalar, tendo em visto que o custo das complicações já estaria no "pacote".<br><br>Desvantagens: tendência a evitar procedimentos de maior risco (mesmo que apropriados) e limitação para a avaliação de desfechos pós-alta. |
| Pagamento baseado em valor | Pagamento por linha de cuidado (remuneração do profissional proporcional ao valor entregue ao paciente)<br><br>Vantagens: modelo que alinha os interesses do paciente, do médico e das operadoras/serviços de saúde. A remuneração do profissional depende do quanto paciente irá utilizar do sistema de saúde (ou seja, quanto melhor o desfecho a longo prazo, menor o custo para o sistema de saúde, maior o valor que irá para a remuneração do profissional).<br><br>Desvantagens: complexidade na aplicação de acordo com as ferramentas atualmente disponíveis. |

Quadro 3. Barreiras e possíveis soluções para implementação do Cuidado em Saúde Baseado em Valor

| **Barreiras** | **Possíveis Soluções** |
|---|---|
| Fragmentação do cuidado por especialidade médica | Criação de times multidisciplinares integrados em linhas de cuidado |
| Falta de mensuração de desfechos que importam para os pacientes | Utilização de desfechos centrados no paciente (se possível, reportado pelos mesmos) e comunicar de forma transparente (sem feedback, não há estímulo para medir e não há melhoras guiadas por métricas) |
| Dificuldade em definir custos no nível do cuidado do paciente | Separar custos por atividade e por tempo dedicado e usar mesmo padrão durante seguimento para conseguir avaliar impacto das ações de melhoria (considerar dados de custos mais como parâmetros para comparações relativas de mudanças ao longo do tempo do que para avaliações pontuais) |
| Cultura atual do pagamento por serviço (do inglês, *fee for service*) | Incluir o prisma do valor em saúde nas decisões médicas e que haja recompensa pela entrega de bons desfechos de forma eficiente (entrega de valor em saúde) |
| Falta de confiança e transparência nas medidas de desfecho e custos | Utilizar meios de obter dados diretamente do prontuário seja por ferramentas padronizadas entre as instituições e/ou por empresas intermediárias que fazem acompanhamento das métricas de valor em saúde de forma independente. |
| Falta de informação do paciente para decidir por cuidado de melhor valor | Apresentar de maneira pública os resultados de cada serviço (procedimento ou linha de cuidado) para que paciente possa escolher de acordo com a melhor proposta de valor apresentada. |
