# Supplementary material for: Cuidado em Saúde Baseado em Valor na Cardiologia: Como Integrar uma Visão mais Abrangente na Decisão Médica?
Source: Arq Bras Cardiol. 2025 Mar 17;122(2):e20240668. [Article in Portuguese] doi: 10.36660/abc.20240668 (PMC12058157; doi:10.36660/abc.20240668)
Supplement: Translation

# Translation: Value-Based Healthcare in Cardiology: How to Integrate a more Comprehensive View into Medical Decision-Making?

**DOI:** 10.36660/abc.20240668i

**Authors:** Pedro Gabriel Melo de Barros e Silva, Valter Furlan, Renato D. Lopes, André Volschan, Paulo Cesar Pereira de Souza, Carisi Anne Polanczyk

**Affiliations:** 1 Brazil Brazilian Clinical Research Institute, São Paulo, SP – Brazil; 2 Brazil Hospital do Coração (HCOR), São Paulo, SP – Brazil; 3 Brazil Hospital Samaritano Paulista, São Paulo, SP – Brazil; 4 Brazil Centro Universitário São Camilo, São Paulo, SP – Brazil; 5 Brazil Hospital Ipiranga, São Paulo, SP – Brazil; 6 Brazil Hospital Paulistano, São Paulo, SP – Brazil; 7 USA Duke Clinical Research Institute - Duke University Medical Center, Durham, NC – USA; 8 Brazil Hospital Pró-cardíaco,Rio de Janeiro, RJ – Brazil; 9 Brazil Instituto Américas, Rio de Janeiro, RJ – Brazil; 10 Brazil Faculdade de Medicina - Universidade Federal do Rio Grande do Sul, Porto Alegre, RS – Brazil; 11 Brazil INCT para Avaliação de Tecnologia em Saúde - Hospital Moinhos de Vento, Porto Alegre, RS – Brazil

**Keywords:** Quality of Health Care, Value-Based Health Insurance, Cardiovascular Diseases

## Abstract

Value-based healthcare is a patient-centered concept that provides a broad view of all parties involved in the care process. This model is designed to achieve the best possible patient outcomes while maintaining cost sustainability. In its classic description, value-based care is the relationship between outcomes that matter to the patient and the costs required to achieve them. Later, other variables were considered in this equation, such as appropriateness and user experience, to make this model more replicable. With a view that aligns the interests of patients, physicians, and payers, value-based healthcare has great potential to improve medical practice sustainably. After more than a decade of discussions and publications, putting this concept into practice is still challenging and dynamic. Value-based healthcare has gained importance and should become a transformative model for health ecosystems. Medical compensation aligned with these principles appears to be a promising path toward such transformation. Nevertheless, the application of this concept depends on a more straightforward definition of the health value parameters in different clinical scenarios. Cardiology is a specialty that usually has a solid evidence base for medical decision-making and this generates great potential for the application of health value elements. In this article, the authors outline a review of the main concepts of value-based healthcare and its potential applications in cardiology.

## Introduction

The concept of Value-Based Healthcare (VBHC) was formally introduced in the early 2000s and is about offering the best possible outcome to the patient in an economically sustainable way, focusing on reducing waste.^1 - 3^It is a conceptual basis for restructuring health systems with the overarching goal of providing care in accordance with the values that matter to patients and that are also aligned with the interests of all the parties involved.^1 - 3^Given the steady increase in health spending without evidence of outcome improvement for the population, strategies are needed to stimulate the generation of better results with the existing resources and within the capacity of the health system itself.^4 , 5^Although this model continues to be poorly understood and measured in medical practice, its application has already yielded effective results.^6 - 10^However, some aspects still need to be considered or improved to enable a large-scale application of VBHC.

This article will discuss the value of health and its practical application in cardiology, in addition to outlining a model to put the concept into practice.

### What is value?

The concept of value is somehow abstract but is used commonly in decision-making in our daily lives. From the perspective of economic sciences, “value” is the propensity to pay for something, i.e., when the benefit received by the customer exceeds the cost of the product or service.^11 , 12^In health, the benefit must be centered on the patient and essentially involves a better quality of life, reduced disease “burden”, and/or increased survival. The cost should be calculated based on the consumption of patient care resources, which can be assessed globally (direct and indirect costs) or specifically (direct cost of an intervention to be performed). Costs can also be calculated from different perspectives (patient, health plan).^13^The relationship between the patient’s benefit and the cost of care is the basis for the rationale of “value” in health.

### The original concept of value in health

The concept of “value in healthcare” was presented more objectively in publications by Harvard Business School Professor Michael Porter.^1 , 2^In the original equation on value-based care ( Central Illustration ), the concept can be interpreted as follows: the better the outcome from the patient’s perspective and the lower the cost of the medical action, the greater the value offered.^2^In addition to medical care, this intuitive concept of “value” is applied to various everyday life decisions, such as purchasing a product or hiring a service, because the better the result of the acquisition (satisfying the existing needs in a long-lasting way) and the lower the cost, the better the application of resources and, consequently, the greater the value attributed to the choice.

Although simple and intuitive, some points should be considered when applying this concept to healthcare activities. The concern that the use of the value in health formula could reward poor outcomes that are achieved at low cost is pertinent and should be considered carefully when implementing this paradigm.^1 - 3 , 12^Thus, the two variables in the equation should also be considered separately and not only collectively because there is no universal value metric that is based on the relationship between the two variables (outcome/cost) and that can be applied to all health decisions.^1 - 3 , 14^Furthermore, this concept of value was created to be applied only when there is no penalty for the relevant clinical outcomes, i.e., lower costs generate real value when the outcomes obtained by patients are maintained or improved.^1 - 3^

#### Outcomes

Outcomes represent health impacts perceived by patients and generated by coordinated processes.^14 - 17^Although measuring process indicators (e.g., door-to-balloon time in Acute Myocardial Infarction) is an important action in improving care, this type of metric will translate into value in health only if it results in better indicators from the patient’s perspective, such as greater survival, reduced complications, and improved quality of life. Process indicators must be pursued to ensure quality of care and tactical planning of services, but they should not be considered as the final objective in health deliveries. In this sense, outcomes vary according to the disease and the individual. It is important to seek outcomes that have greater relevance and validity for population health, and that can also be evaluated objectively and hierarchically (e.g. in prostate surgery, the hierarchy may be survival, incontinence, and erectile dysfunction, although this order may vary between individuals). Outcomes must represent sustained benefits for the patient and must be relevant to other parties in healthcare.^14^

In addition to the clinical outcomes themselves, several actions can influence the cost of healthcare and/or the experience of individuals, but not necessarily the patient’s clinical outcome. Although these coordinated processes do not change the clinical outcome, they can reduce costs and waste and could increase the value of health as long as they do not compromise survival and quality of life.

#### Costs

In health value analysis, when the outcomes are not compromised (numerator), any action that implies lower costs (denominator) consequently generates greater value.^2^In this sense, planning care processes that streamline the flow of care with well-defined metrics for continuous improvement, even if they do not change the outcome, can improve efficiency (and, at some point, the experience of individuals) and, consequently, reduce costs and increase value in health.^18^The cost is initially calculated directly. However, indirect costs can be included in the value denominator in the health formula for a more comprehensive assessment.

In Brazil, it is still very challenging to estimate the real costs of healthcare. This often leads to situations where a specific procedure (which should have a similar cost in different units) costs a different amount (the amount changes according to the type of health plan or hospital negotiation). The financial logic of most national institutions is based on structural units (intensive care unit, emergency, hemodynamics), with values being distributed by apportionment or absorption techniques, leaving out the patient’s trajectory or a specific health condition. Micro-costing techniques allow us to estimate the real cost of health services.^19 , 20^This distortion is reflected in both overfunding in health (the fund is way higher than the cost) and underfunding in health (the fund is lower than the cost of care). These two extremes create a situation of uncontrolled costs that drain the sustainability of the health system.

## The concept of appropriateness

In addition to outcome and cost, two other variables have been discussed in this equation: appropriability^21 , 22^and patient experience.^22^

Appropriateness (or relevance) indicates whether a medical action has an adequate scientific basis to improve the patient’s outcome.^21 , 22^In a categorical analysis (yes or no), this variable would enter the formula as 1 or 0, i.e., if it is appropriate (improves outcome), quantification is done according to the classic formula (impact on outcome/cost). However, if it is not appropriate, the result will always be zero (there will be no value). This concept is already considered in cost-effectiveness analyses. If an action is not efficient or effective, there is no reason to evaluate the cost because the action will not be cost-effective, no matter how small the cost is.^23^

However, in several medical decisions, there is a “gray” zone in which it is not possible to assign a value of 0 or 1 (especially in population parameters in a heterogeneous clinical situation).^24^Given the existence of uncertainty regarding appropriateness, this variable is especially useful when there is sufficient evidence to classify a decision as appropriate or inappropriate. This is a simple model that can be applied objectively to actions that are not appropriate and do not generate value (since the same outcome would be achieved at “zero cost”) even when the patient has a good outcome at “low cost”.^23 , 24^Identifying irrelevant actions helps reduce the waste caused by overdiagnosis and overtreatment actions ( Table 1, supplementary material ). In any case, even in “gray” zone situations (e.g. scenarios that are not the same as the ones tested in a clinical trial), having a value in health view will lead physicians and/or managers to seek more elements to make more effective decisions for patients because they know these scenarios involve uncertainty in terms of appropriateness and value.

Despite the rationale for considering appropriateness “uncertainty” in some situations, international recommendations (US, Europe) say that a medical action that cannot be classified as appropriate should already be intrinsically considered a form of waste and poor practice.^21 - 27^This is based particularly on the potential consequences of overdiagnosis and overtreatment, which can be significant and include everything from the psychological and behavioral consequences of patient stigmatization to physical harm/side effects of unnecessary tests or treatments that impair quality of life and may even shorten the individual’s survival.^21 - 27^In addition to the possibility of worsening outcomes, excessive actions increase individual financial costs and generate waste of resources and opportunities in the health system. Although it conceptually makes sense not to perform a procedure whenever its real benefit is questionable, care should be taken not to generate the opposite consequence if the procedure is not performed, i.e., underdiagnosis or undertreatment. Even though finding balance is challenging, applying the concept of value in health is a rational way to achieve greater maturity in medical decision-making and healthcare policies.

## Other goals in healthcare

In addition to outcome, cost, and appropriateness, other metrics should also be considered as goals in value-based care. Although including the patient (“client”) experience in the value formula has a great appeal from the healthcare market point of view, this could add in more “noise” rather than information about the clinical result itself.^1 , 2 , 15^This does not mean that the experience does not add value to patient care. Nevertheless, it is another type of value (e.g., user perception that includes variables such as hospitality, reception, and auxiliary services of the healthcare unit, among others). Despite representing something different from the outcome itself, the patient’s experience in healthcare should be valued like the other elements of the VBHC, especially when searching for what matters to the patient and that can be measured reliably.^28^

Once this variable (patient experience) is added to the valuation of services, it should be assessed independently of the outcomes. For example, a patient may have undergone a high-cost treatment that did not change the outcome because it was an inappropriate therapy (overtreatment) and, even though the experience was good, there was no value in health (the opposite is also true, for example, an appropriate treatment that improved the clinical outcome at a low cost generated high value in health even if the patient was not satisfied with the way they were cared by the team). Experience issues must be identified and managed in a specific way (i.e., differently from clinical outcomes).

Finally, other items have been added to the primary objectives of healthcare, since the classic health value formula that was based only on outcomes and costs.. The so-called triple aim indicates that the health system must focus not only on patient outcomes at a sustainable cost but also on user experience.^22^This concept was expanded to include a quadruple aim, in addition to considering the patient’s experience, it also includes that of the healthcare professional. Therefore, in addition to the best patient outcome at the lowest possible cost, the best experience is also sought for both the patient and the healthcare professional.^25^

More recently, health equity has also been included as one of the primary objectives, creating the so-called quintuple aim.^29^In this case, the aim is to reduce healthcare access and outcome inequality among different populations, removing geographic, financial, or cultural barriers in underprivileged communities ( Central Illustration ).^29^These concepts can be better applied in health networks. Health networks manage a large and diverse population and can act more robustly in the equity of care.^29^In this sense, there must be integration of care provision in multiple health units not only to reduce costs (e.g., avoid duplication of efforts, delays and other inefficiencies in the health process), but also for real population management and, ultimately, improved equity.^30^

## Applying the concept of value in health to cardiology

The current challenge has been to transform conceptual aspects into practical actions, providing greater understanding to each of the “players” in this new model. Although VBHC is patient-centered and depends on team-based care for the implementation and success of this model,^31^some decisions made by cardiologists can be guided by the principles of value in health. Below are examples of how to apply these concepts to coronary heart disease:

## Acute coronary syndrome (ACS)

Although the value of the health model is often applied to therapeutic interventions (e.g. surgeries),^32 , 33^this concept can also be considered in other medical actions, such as the diagnosis of acute myocardial infarction. The biomarker of choice for this diagnosis is high-sensitivity troponin.^34^However, CKMB continues to be routinely requested concomitantly with troponin in many services.^35^Using the expanded value formula, adding routine CKMB measurement does not add value to patient care as it has no positive impact on any of the variables: 1) it is not considered an appropriate test, 2) it does not improve patient outcome, 3) it increases cost, 4) and in terms of patient experience, an additional test would not improve it. Thus, although no numbers are used in this formula, it can be seen that this medical action has no value.

In a time-sensitive disease care setting such as ST-elevation ACS, evaluation by a specialist physician would be the “gold standard” for diagnosis and management. However, it is not feasible to have a cardiologist available in all emergency rooms to treat these patients. Therefore, the support of a specialist via telemedicine would be an attractive strategy to ensure the support of a cardiologist in a greater number of units. This model has been associated with improvements in the therapy of patients with ACS and, consequently, improved outcomes.^36 , 37^Although specific evidence on telemedicine in this ACS scenario still lacks validation in randomized clinical trials, this action was designed to deliver value in health, i.e. improve the outcome at a sustainable cost.

Value in health begins with a focus on quality (outcome) and several randomized studies have consistently shown that interventions that improve quality generate greater use of evidence-based therapies and some with the power to demonstrate better outcomes.^38 - 41^Specifically in ACS, a national study called BRIDGE-ACS^38^randomly evaluated the impact of care quality interventions. Although not designed with the power to assess the impact on clinical outcomes, BRIDGE-ACS demonstrated significant improvement in the use of evidence-based therapies in ACS.^38^

Value-based care has the improvement of care quality and clinical outcomes for patients as its primary objective. However, value delivery must also focus on the cost involved in achieving this desired outcome, i.e., both outcomes and costs must always be evaluated. In a national experience, the use of international tools for quality care actions was associated with improvements in the treatment and outcomes of patients with ACS.^42^However, it was necessary to understand whether these actions would also have an impact on the costs. When analyzing hospital costs and readmission rates of patients with ACS, hospital costs were better controlled and there were fewer readmissions to the hospital under the quality care improvement actions when compared to the cost of similar patients in other units.^43^As demonstrated in this example in which quality and cost metrics were measured,^42 , 43^in a program aimed at improving the quality of care in ACS, it is expected that, in the different lines of cardiology care, since there are better outcomes (fewer complications), the cost in healthcare will also be lower and there will be fewer readmissions.

## Myocardial revascularization

In patients adequately indicated for myocardial revascularization, i.e., when there is evidence of better outcomes for the patient with this strategy, it is important to evaluate which procedure would bring the best value in health in a sustained manner.^44^

In situations where there is an appropriate indication for myocardial revascularization, whether percutaneous or surgical, one should also evaluate how to improve cost and patient experience without compromising clinical outcome. In general, actions that improve quality will increase value not only because of their impact on better outcomes but also because better quality in myocardial revascularization is associated with lower costs.^44 , 45^Thus, in addition to actions focused on achieving the best clinical outcome, initiatives to improve efficiency (e.g., reduce hospital stays after cardiac surgery and angioplasty) also add value to the care process, since they reduce costs without compromising clinical outcomes (and may even improve patient recovery).^46 , 47^Initiatives such as same-day discharge protocols after uncomplicated angioplasty^46^and enhanced recovery after surgery (ERAS),^47^although they do not yet have robust evidence for broader recommendations, are actions that aim to offer value because they seek to at least maintain clinical outcomes and, at the same time, reduce costs and improve the patient experience.

## Other examples

Whatever the clinical situation (intervention, chronic disease), value delivery depends on an integrated care network in which care is provided by a dedicated multidisciplinary team assuming responsibility for the complete cycle of care for a specific condition. This covers outpatient, inpatient, and rehabilitation care, in addition to support services.^30 , 48 , 49^The adoption of the best practices should be guided by evidence-based guidelines^50 , 51^and, in addition to the examples of value in health in ACS and myocardial revascularization, other examples can be found in cardiology that allow for a broader implementation of this new model. In this full application of value-based care, a compatible form of compensation is necessary because the current compensation models are not aligned with the principles of value-based care.^5 , 52^

## Traditional compensation models

Currently, different compensation models ( Table 2, supplementary material ) are used in different health systems, many of which coexist within the same ecosystem.^53 - 55^

• The oldest and most prevalent form of compensation in health systems, including Brazil, is fee-for-service, which focuses on reimbursement based on the production of activities and, therefore, does not align the interests of all the parties involved ( Table 2, Supplementary Material ). In this model, when using a health plan, both the user (patient) and the service provider (health professional or institution) establish a relationship in which deliveries are linked to the product (or service provided) and not to the actual gain (or benefit) in health. In other words, regardless of the health results or relevance of the service, there will be a fixed payment, which encourages production in greater quantities, even if this does not effectively generate value in health. This model includes payment methods usually referred to as compensation per unit of service or open account, and compensation for packages of procedures and hospital daily rates, with the essence being payment according to the number of individual procedures. One of the main disadvantages of this model is that it encourages the use of services in groups with questionable benefits, especially those that provide higher profit margins which, therefore, can negatively affect the quality of healthcare and the sustainability of the system. In the literature, this is currently the model most associated with the unnecessary increase in healthcare costs.
• The “bundle” payment model consists of grouping payments for a specific health condition, procedure, or surgery considering a longitudinal follow-up period. May or may not consider risk sharing between payers and providers. There are specific “bundles” (e.g. for acute cardiac conditions, post-surgery, or transcatheter intervention), with follow-up generally lasting 30 to 90 days. “Bundle” payment can also generate higher financial returns through volume and it also encourages the teams to perform procedures in situations with a lower probability of complications when the costs of any complications are assumed by the teams themselves. This represents a potential risk for performing procedures in situations with less need (or appropriateness). Additionally, teams would tend to avoid procedures that, despite being appropriate, have a considerable risk of complications (which can generate greater limitation of care in groups with more severe diseases).
• In the per capita or capitation model, health system providers receive a fixed compensation for partial or full healthcare, for a defined population. The capitation model ensures the necessary care for those who need it, regardless of whether or not that person uses the service. The value is established per person included in the program (per capita) and for each period. Therefore, the compensation amount is based on the expected average utilization of each patient (planned compensation should vary according to age, race, sex, region, and, mainly, medical history). These models can have their relationship bases considering objective deliveries per service provided (e.g.: number of hypertensive patients with blood pressure measured or colonoscopy performed on individuals over 50 years old) or per broader metrics that refer to population health indicators (mortality due to myocardial infarction, hospitalization due to heart failure). It should be noted that this modality presupposes knowledge of the health risks associated with coverage of the defined population, as well as the counterpart in terms of costs.^56^One of the major difficulties in advancing the capitation model is that its success depends on a more prolonged chain of analysis (with time and variables to be considered) since there is a potential risk of restricting access to healthcare to reduce costs in the short term and, consequently, postponing the solution of the patient’s problem to a later date (which can increase costs in the long term). Therefore, the safety and sustainability of this model depend on the effective management of patient care information, outcomes, and costs to prevent, mainly, underdiagnosis and undertreatment resulting from potential short-term access restrictions.

## Value-based compensation

Value-based payment or compensation is a healthcare delivery model in which providers, including hospitals and physicians, are paid based on patient health outcomes ( Table 2, Supplementary Material ). In this type of agreement, providers assume responsibility for healthcare deliveries to patients. Value-based compensation provides a more appropriate incentive than other current models since its principle is to provide the best patient outcome at the lowest possible cost.^54^Consequently, unlike other models in which the financial return is linked to a greater number of services (fee for service) or reduced higher cost/risk procedures (bundles, capitation), value-based compensation is based primarily on patient outcome and, consequently, the interests of all the parties involved are aligned.^55^

When using a value-based compensation model, it is important to understand that part of the outcome depends on the patient (e.g., medication adherence) and not directly on the healthcare professionals. In any case, patient adherence is directly associated with health education and, in this case, having a model that performs comparative analyses of the results between professionals or teams can improve the result as a whole without distorting value-based compensation.

In this integrated and coordinated model, healthcare providers work as a networked team to provide the best possible care at the cost necessary to achieve it. In this way, in addition to the individual result, the care line team shares the risk and financial return. The structuring line of the value-based payment model should focus on the adoption of best medical practices, based on scientific evidence and international guidelines, with a long-term time horizon of results.^54 , 55^

Another alternative payment model is the Global Budgeting or Budget Transfer, which is a form of prospective compensation, in which the health establishment annually estimates its spending needs and presents them to the financing entity. In return, it commits to meeting performance targets, based on efficiency and quality metrics of service provision.^57^One of the main issues related to this model is the complexity of its implementation and measurement of indicators when multiple payers of services are linked to the establishment.

## Current status of compensation models

The current reforms underway in health systems around the world aim to reduce the scope of the current fee-for-service model through alternative compensation models.^53 - 58^In the international context, specifically in the private health insurance and plans market, new products have emerged, such as what has become known in the US market as Value-Based Insurance Products.^58^In general, both the Shared Savings Program (SSP) initiative and the Bundled Payments can be grouped within the category of new compensation and risk-sharing models that emerged from international reforms and are guided by values of quality and efficiency.

## Value-based medicine culture

The implementation of a payment model that is completely different from the current one, in a complex system such as healthcare, depends not only on the actions of healthcare managers, but also on a cultural change among physicians, patients, and society in general.

Value in healthcare is derived from measuring clinical outcomes achieved and the cost required to obtain them. This concept should not be restricted to the physician and health manager but should be part of the patient’s decision. Just like when purchasing a product or hiring a service, a healthcare “client” should also seek information on the results of a hospital or physician. In the current healthcare model, professionals and hospitals are often chosen based on cost (low or high) and/or social recognition, as if they were synonymous with value in healthcare, which does not necessarily ensure the concept described.

As part of an information asymmetry, patients have little information about fundamental aspects, such as the appropriateness of medical actions, clinical results (for the physician and hospital), and avoidable costs in healthcare. The availability of this information, in addition to benefiting the patient, can also lead to a reduction in healthcare costs, which, ultimately, could increase patient access to the healthcare system and increase the average compensation of professionals in a value-based payment model.

One of the major difficulties in implementing these more adaptive models is ensuring the security and veracity of the information reported by providers (hospitals, clinics, professionals). This limits the application of this concept only to more controlled health systems (e.g., verticalized networks).^59^

Following the precepts of value-based care, accountable care organizations (ACOs) have emerged. In these organizations, the patient and providers are true partners in care decisions (patient-centered).^60^In this model, there is an emphasis on coordinating care with the sharing of clinical and claims data among team members and also with payers so that compensation is linked to improvements in results (such as hospital readmissions, adverse events, patient engagement, and population health indicators). For it to be truly considered a value-based model, it must have the best outcome for the patient as a principle and not pursue economic results only. Methods for the uniform assessment of outcomes have been published and applied, such as ICHOM (International Consortium for Health Outcomes Measurement), which have been useful in guiding lines of care.^61^Models for collecting data directly from patients for both outcomes (PROMs, Patient-Reported Outcomes Measures) and experience (PREMs, Patient-Reported Experience Measures) are recommended.^62^

In any case, whether applied on a larger or smaller scale, the culture of value in health must be present in the design of medical services, based on actions that provide the best outcome through a coordinated process of care quality and safety. When the best outcomes for specific health conditions or populations have been defined and agreed upon, these processes must be evaluated to see if they can be streamlined and, consequently, reduce costs to deliver greater value in health. One of the initial steps for practical application would be to recognize the barriers and continuously seek solutions that are adapted to each reality ( Table 3, Supplementary Material ).

## Applying the concept of value in health - A proposal

In many locations, the VBHC model has been implemented as a pilot project to allow for initial adjustments before expanding. In these services, teams usually have good communication, good knowledge of evidence-based medicine, reliable data, and good adherence to protocols.^63 - 65^The following are some fundamental elements of a value-based system ( Figure 1 ):

**Figure 1:** – Fundamental elements of value-based healthcare (framework).

- Organize care according to medical conditions, i.e., lines of care for the medical conditions of patients or segments of the population;
- Provide electronic medical records that allow information sharing among all providers in the care line;
- Care should be based on a “team” model with sharing of patient data for a coordinated process that reduces redundant care (and associated costs) and includes easily measured outcomes (in value-based health models, emergency, primary, or specialized medical care does not occur in “silos” but rather in an integrated, patient-centered line of care);
- Keep the patient under continuous monitoring, providing adequate coordination and delivery of care;
- Define the appropriateness of medical actions according to the medical situation based on the best available evidence;
- Once appropriateness has been defined, evaluate the metrics to be measured (outcomes that matter to the patient according to the type of disease and proposed treatment);
- Have metrics not only for clinical outcomes, but also for costs, for each patient, and evaluate opportunities to improve efficiency and patient experience;
- Evaluate outcome indicators regularly and share them with the entire healthcare team;
- Keep the focus on patient safety (avoiding harm caused by care) and always seek to improve clinical outcomes;
- Finally, be transparent with “consumers” so they can be partners in building VBHC.

The implementation of value-based payment must be done following the above premises and, although there is still no universal formula, payment models should reward the best outcomes (relevant and sustained) and efficiency of care (lowest possible cost as long as it does not compromise the outcome). From the point of view of the health system, one way to achieve greater efficiency would be to integrate a healthcare network in which the provision of care is organized hierarchically. The rationale behind this integration is that a system with several centers that handle highly complex cases will generate an environment with lower efficiency, higher costs, and, consequently, lower value. In addition to greater efficiency and lower costs in a hierarchical integrated network, the concentration of care lines in reference care units allows the quality process to develop more fully and, therefore, better outcomes are expected (which would further increase the value delivered to patients).

## Conclusions

The shift from the current system to a value-based model is challenging, although the transition is happening in different parts of the healthcare ecosystems. This is a process that tends to be gradual, but it must be scaled up quickly, especially for chronic patients. Value-based cardiology is an international trend that aims to increase quality care at the lowest possible costs. This could be the way to help more people live healthier lives in a sustainable healthcare system.

## *Supplemental Materials

For additional information, please click here.
